# Maternally Derived Immunity Extends Swine Influenza A Virus Persistence within Farrow-to-Finish Pig Farms: Insights from a Stochastic Event-Driven Metapopulation Model

**DOI:** 10.1371/journal.pone.0163672

**Published:** 2016-09-23

**Authors:** Charlie Cador, Nicolas Rose, Lander Willem, Mathieu Andraud

**Affiliations:** 1 Swine epidemiology and welfare research unit, French Agency for Food, Environmental and Occupational Health & Safety (ANSES), Ploufragan, France; 2 Centre for Health Economics Research & Modeling of Infectious Diseases, Vaccine and Infectious Disease Institute, University of Antwerp, Wilrijk, Belgium; 3 Université Bretagne Loire, Rennes, France; University of Minnesota College of Veterinary Medicine, UNITED STATES

## Abstract

Swine Influenza A Viruses (swIAVs) have been shown to persist in farrow-to-finish pig herds with repeated outbreaks in successive batches, increasing the risk for respiratory disorders in affected animals and being a threat for public health. Although the general routes of swIAV transmission (i.e. direct contact and exposure to aerosols) were clearly identified, the transmission process between batches is still not fully understood. Maternally derived antibodies (MDAs) were stressed as a possible factor favoring within-herd swIAV persistence. However, the relationship between MDAs and the global spread among the different subpopulations in the herds is still lacking. The aim of this study was therefore to understand the mechanisms induced by MDAs in relation with swIAV spread and persistence in farrow-to-finish pig herds. A metapopulation model has been developed representing the population dynamics considering two subpopulations—breeding sows and growing pigs—managed according to batch-rearing system. This model was coupled with a swIAV-specific epidemiological model, accounting for partial passive immunity protection in neonatal piglets and an immunity boost in re-infected animals. Airborne transmission was included by a between-room transmission rate related to the current prevalence of shedding pigs. Maternally derived partial immunity in piglets was found to extend the duration of the epidemics within their batch, allowing for efficient between-batch transmission and resulting in longer swIAV persistence at the herd level. These results should be taken into account in the design of control programmes for the spread and persistence of swIAV in swine herds.

## Introduction

Swine Influenza A Viruses (swIAVs) are widespread in pig-production units. Three main subtypes (H1N1, H1N2 and H3N2) are circulating worldwide [1–3] and have evolved in different lineages with genetic components from both avian and human viruses. The co-circulation of different subtypes and strains [4,5] increases the probability of co-infections, which, in turn, may lead to the emergence of reassortant viruses [6–8]. The new viruses could potentially be more pathogenic for the animals and/or transmissible to humans [9,10]. Therefore, understanding the dynamics of influenza viruses in swine production units is pivotal to both animal- and public-health perspectives.

Endemic forms of influenza infections are increasingly reported in swine production units [4,11,12]. Factors responsible for these repeated infections in successive batches include husbandry practices and suspected adverse effects of maternally-derived antibodies (MDAs). MDAs were shown to significantly reduce the clinical expression in young animals while not fully preventing swIAV transmission [13–16]. This may lead to a silent spread of the virus in the first weeks of age, which could partly explain the recurrence of epidemics after passive immunity waning.

Modelling approaches have been successfully used to investigate within-herd transmission and control measures for other viruses or bacteria affecting pigs considering the batch structure of pig herds [17–22]. The contact structure within a population is known to influence transmission dynamics of pathogens [23]. However, Dorjee et al. [24] stressed the limited knowledge of influenza transmission at pig farm level, which could be a key to manage the risk of emergence of novel influenza viruses in human population. To date, a few mathematical modeling studies have been focusing on swIAV dynamics of infection in pig herds. Reynolds et al. [25] recently developed a deterministic model representing swIAV dynamics in US breeding and finishing herds with large population sizes. Assuming a constant indirect transmission between the different farm buildings, the authors showed that the virus was able to persist in the breeding farm. The assumption on indirect transmission is not appropriate in farrow-to-finish pig farms organized in batch-rearing systems. Indeed, farrow-to-finish pig herds are usually segregated in specific sectors according to their physiological stage [26] with no or relatively low number of contacts between the different sectors. Moreover, within each sector, each batch is usually independently managed to prevent mixing of animals with different health and immune statuses [27–29]. Farrow-to-finish systems in Europe are nevertheless strongly associated with the issue of swIAV persistence. In these systems, farrowing occurs at regular intervals leading to a regular reintroduction of susceptible piglets in relatively small subpopulations in the nursery, the central point between breeding sows and growing pigs. The batch-rearing management induces also a specific contact structure between the small metapopulations. A stochastic approach is therefore more suited to represent swIAV transmission process within a typical farrow-to-finish pig herd [23,30]. More recently, Pitzer et al. [31] developed a stochastic model to evaluate the impact of herd size on swIAV persistence at the herd level. The impact of MDA protection on swIAV persistence was briefly considered but the characteristics of the infection dynamics associated to different levels of MDAs in the population remain largely unknown.

In this paper, the role of MDA-positive piglets in swIAV spread and persistence in farrow-to-finish pig production units is investigated using a stochastic metapopulation model. The model structure, parameters and assumptions are presented using the ODD protocol (Overview, Design concepts, and Details) developed by Grimm et al. [32,33]. Next, the uncertainty analysis to assess the impact of unknown parameters on model outputs is described. Finally, the modeling results are presented and discussed.

## Model Description

### Purpose

A mathematical model simulating the population dynamics within a farrow-to-finish herd and representing animal housing facilities has been coupled with an epidemiological model of swIAV transmission. The resulting stochastic event-driven model was used to assess whether passive immunity could favor within-farm swIAV persistence.

### Model’s entities, state variables and scales

#### Population

A farrow-to-finish herd comprises typically two subpopulations of animals—breeding sows and growing pigs—considered together in the model and leading to the representation of a metapopulation, subdivided in a set of collectives, called batches hereafter. As swIAV infection is known to have a limited clinical impact [1], no disease-related mortality was considered in the model so the number of individuals per batch (breeding sows or growing pigs) is assumed constant. Batches are characterized by the state variables corresponding to the batch identity number, physiological stage, exact location in the herd (room number) and the distribution of the animal health states.

#### Environment

**Fig 1:**
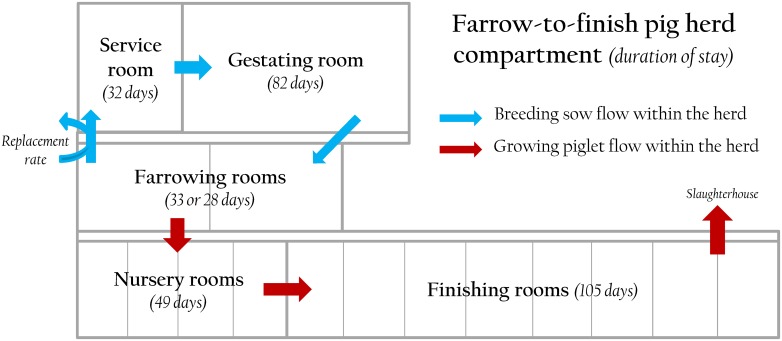
Facilities modelled in the farrow-to-finish pig herd.

According to their physiological stage, animals evolve through five types of facilities: the service, gestating and farrowing facilities for breeding sows; and the farrowing, nursery and finishing facilities for growing pigs (Fig 1). Farrowing, nursery and finishing facilities are divided into several rooms, managed according to an all-in-all-out strategy, *i*.*e*. all animals in a batch leave the facility at once and enter an empty room simultaneously; each batch is therefore managed independently with limited relationships through environmental components (*e*.*g*. air-flow or farm material). In service and gestating facilities, all the batches are gathered in unique rooms. The two subpopulations (sows and piglets) physically interact exclusively in farrowing rooms.

#### Time scale

The model was built in continuous time (event-driven model). Considering the fast-acting progression of swIAV in swine population, simulations were run over a 4-year period to analyse the swIAV endemic persistence over time after introduction in an infection-free herd. Parameters are defined on a daily basis.

### Process overview and scheduling

The modeling time-steps are event-driven with two types of processes: events related to the population dynamics (movements between facilities) and health state transitions.

#### Population dynamics process

The parameters governing the population dynamics are depicted in Table 1.

**Table 1:** Parameter values used in the metapopulation dynamics model reared in a 7-batch rearing system (from: [34,35]).

Parameter description	Value
Duration of a sow reproductive cycle *(days)*	147
- Duration in service room *(days)*	32
- Duration in gestating room *(days)*	82
- Duration in farrowing room *(days)*	33
Duration of a growing pig cycle *(days)*	182
- Duration in farrowing room *(days)*	28
- Duration in nursery room *(days)*	49
- Duration in finishing room *(days)*	105
Interval between two successive batches of sows and pigs *(days)*	28
Culling rate after weaning for sows *(%)*	16.4^a^
Average number of piglets per litter	11.5
Number of sows per batch	24
Number of piglets per batch	276

Assumptions are based on current knowledge on swIAV.

^a^16.4% of breeding sows are culled at each reproductive cycle corresponding to a culling rate of 38.5% per year with 2.35 reproductive cycle per sow per year (reproduction performance values, [34]).

**The breeding sow cycle:** The sow reproductive cycle (147 days) is made of three different physiological stages to which correspond three different types of facilities. At introduction or after weaning, gilts and sows are moved to service room where they are inseminated 4 days later and remain in this room up to 4 weeks after insemination. Then, batches of sows are moved to the gestating room for 82 days until farrowing entrance.

**The lactating stage:** Every 3 weeks, a batch of sows joins the farrowing room 5 days before farrowing for acclimatization (115 days of gestation in total) and gives birth to a batch of piglets. Dams remain with their litter 4 weeks (lactation period) until weaning. At that time, sows are moved back to the service room to begin a new reproductive cycle starting with sow culling and renewal. Piglets are moved to a vacant nursery room.

**The growing pig cycle:** Each batch of weaned piglets occupies a nursery room until 11 weeks of age before moving to a vacant finishing room until their slaughter-age (182 days of age).

#### Epidemiological process

Events representing swIAV infection process are stochastic. The transitions between health states do not depend on the time spent by the animal in the previous health states (Markovian process).

**Fig 2:**
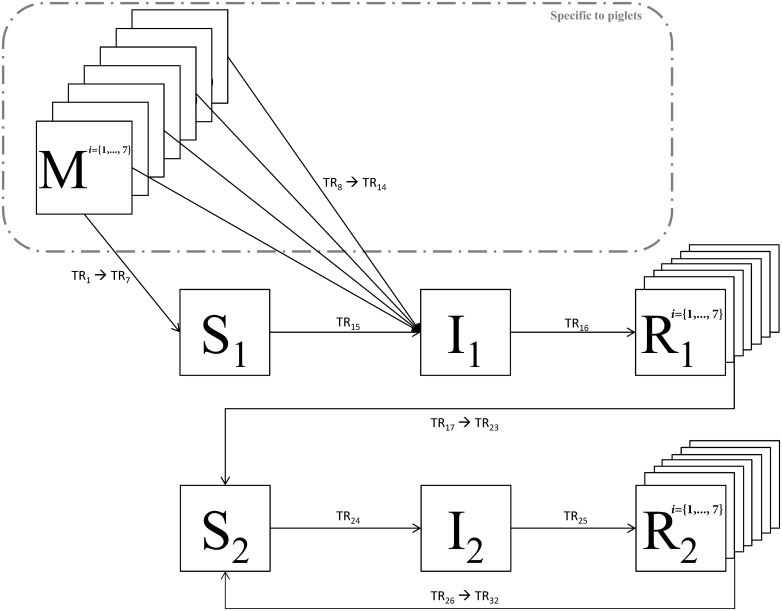
swIAV infection states. *M*: Animals with MDAs; *S*_1_: Naïve animals; *I*_1_: Infected; *R*_1_: Recovered; *S*_2_: Susceptible to reinfection; *I*_2_: Re-infected; *R*_2_: Recovered after reinfection. Full transition rates (TR_1_ → TR_32_) are developed in Equations (Table 3).

The swIAV infection process and the governing parameters are depicted in Fig 2 and Table 2, respectively. The model accounts for both horizontal and/or airborne transmission routes. The different events and their associated transition rates are described in the *‘Submodels’ section*. The duration of passive and active immunity (M and R states, respectively) are assumed to follow gamma distributions modeled using the stage approach [36–38] and the infectious period is exponentially distributed. Each random time step corresponds to the update of the health state of one single individual. Rate equations are then updated to account for the new situation.

**Table 2:** Parameter values used in the swIAV infection dynamics model.

Rate	Event	Sources	Base value	Variation in the uncertainty analysis
*β*_1_	Transmission rate due to infected animals *I*_1_	[16]	2.43^a^	-
*φ*	Susceptibility to reinfection	*Best guess*	0.75	0.50–0.75–1.0
*β*_*air*_	Between-batch transmission rate	*Best guess*	0.1	0.01–0.05–0.1–0.3–0.50–1
*ε*	Susceptibility to infection for piglets having MDAs	[16]	Mean = 0.39	-
*γ*_1_	Recovery rate for infected animals *I*_1_ (days^-1^)	[16]	1 / 6.1	-
*σ*_1_	Immunity waning after the first infection (days^-1^)	*Projected from field observations*	1 / 180	90–120–150–180–240–360
*σ*_*m*_	Maternal immunity waning (days^-1^)	[39,40]	1 / 70	-

^a^ *β*_1_ is 1.41 in the service room [16] as sows are reared in specific conditions (see “Parametrization” section for more details).

### Design concepts

#### Basic principles

A stochastic event-driven batch-based model, ruled by the Gillespie’s Direct Algorithm [23], has been developed in Matlab (MATLAB 2012b, The MathWorks, Inc., Natick, Massachusetts, United States) by coupling the metapopulation dynamic model representing the herd structure and management with the swIAV epidemiological transmission model. swIAV spread in pig farms is mainly caused by pig-to-pig contacts and airborne transmission [41]. Consequently, the local probability of infection for a batch depends on (i) the within-batch infection process occurring by direct contact and (ii) the between-batch infection process transmitted per airborne route. We assume that—within a batch (*i*.*e*. within a room)—all animals have the same contact rate with their infected roommates and are equally exposed to the infectious pressure from infectious animals in neighbouring rooms (all the rooms belonging to the same facility). The epidemiological model is an extended SIR model—Susceptible (S), Infectious (I) and Recovered (R)–also accounting for MDAs and possible reinfection by the same subtype (Fig 2). Indeed, field data suggest a possible reinfection in sows which could explain the observed rise of HI (Hemagglutination Inhibition) titres according to parity [4,5]. Newborn piglets born to immune sows acquire swIAV MDAs by colostrum intake (health state M) providing partial protection towards infection [4,13,15,16].

#### Within-batch interactions

Two within-batch transmission rates, *β*_1_ and *β*_2_, are considered according to the infection-history: *β*_1_ applies to infected animals *I*_1_ and *β*_2_ to re-infected animals *I*_2_ (Table 2). Faster onset of the immune response is assumed for animals already exposed to the virus, due to an immune system memory effect, leading to a shorter shedding period with lower levels of virus shedding. Therefore, a factor *φ* was introduced to reflect the potential impact of the immunity boost in re-infected animals on both transmission efficacy and shedding period ($\beta_{2}=\varphi \beta_{1},\;\frac{1}{\gamma_{2}}=\varphi \frac{1}{\gamma_{1}},\;0<\varphi <1$). In addition, the duration of the immune period after reinfection is assumed longer ($\frac{1}{\sigma_{2}}=\frac{1}{\varphi }\frac{1}{\sigma_{1}}$).

Next, a reduction of susceptibility to infection in MDA-positive piglets is considered [16]. A factor *ε* (0 < *ε <*1) was introduced to represent the increase of piglets’ susceptibility while passive immunity waning. Therefore, the force of infection depends on the proportion of infected pigs and the host immune status.

#### Between-batch interactions

Farrowing, nursery and finishing facilities are divided in several rooms housing distinct batches of animals. Hence, the model accounts for between-batch interactions *via* airborne transmission from neighbouring rooms and the corridor during animals transfer between different facilities. For that purpose, a specific transmission rate *β*_*air*_ was considered. Infectious aerosols are produced by shedding pigs hence the proportion of shedding animals in neighbouring rooms is used as a proxy of the viral load in the air.

Moreover, weaning is an event with interaction between the farrowing, nursery and gestating facilities. Therefore, a transient probability of infection is introduced on weaning-days through airborne transmission between the different facilities.

#### Stochasticity

The transmission model is stochastic with time-steps sampled from an exponential distribution with an expected value determined by the sum of transition rates governing swIAV transmission *(see ‘Submodels’ section)*. Two additional stochastic processes were incorporated using binomial distributions: (i) the transmission process during transfer at each farrowing room entrance or exit; (ii) the renewal process in the breeding herd.

#### Observations

Each time-step corresponds to one event targeting either one individual (health state transitions from the epidemic process) or a batch (facility changes). Daily snapshots of the population are recorded as model output. This matrix comprises the simulation time, the batch identity number, the herd location and the number of animals in each health state. Population level prevalence can then be calculated at any time point. The time to swIAV fade-out (*i*.*e*. first time without shedding animals in breeding sows AND growing pigs) and batch-related characteristics of the epidemic (number of shedding animals, mean duration and variation between batches) can also be deduced from model output.

### Initialization

The model is initialized by assigning 24 susceptible sows in each batch in service room every 3 weeks. The times spent in each facility are calibrated using published data and experts’ opinion, to represent the population dynamics of a realistic farrow-to-finish pig herd (Table 1). One single infectious gilt is introduced once in the service room during the first replacement to initiate the infectious process. We assumed no subsequent introductions of infectious animals. Model initialization was similar for each simulation and no swIAV prevention or control measures are implemented over time.

### Input

The model does not incorporate external or seasonal processes.

### Submodels

#### Force of infection *λ*

As described above, two within-batch transmission rates (*β*_1_ and *β*_2_) and one airborne between-batch transmission rate (*β*_*air*_) were considered in the model. Based on those transmission rates, at each time step *t*, two specific forces of infection *λ* were calculated as follows:

- The within room specific force of infection *λ*_w_(*t*,*r*), at time *t* in room *r* and given by:
  $$\lambda_{w}\left(t,r\right)=\;\frac{\beta_{1}I_{1}\left(t,r\right)+\beta_{2}I_{2}\left(t,r\right)}{N_{r}^{}}$$
  with *I*_1_(*t*,*r*) and *I*_2_(*t*,*r*) being the number of infected and re-infected animals at time *t* in room *r*; *β*_1_ and *β*_2_ their respective transmission rates per day and *N*_*r*_ the total number of growing pigs in room *r*.
- The between-room airborne force of infection *λ*_*b*_(*t*,*r*), considered in nursery and finishing facilities, is calculated from the total prevalence of infected animals at time *t* in neighbouring rooms *r’*:
  $$\lambda_{b}\left(t,r\right)=\beta_{air}\frac{\sum_{r\prime \neq r}^{}I_{1}\left(t,r^{\prime }\right)+I_{2}\left(t,r^{\prime }\right)}{\sum_{r^{\prime }\neq r}^{}N_{r\prime }},$$
  with *I*_1_(*t*,*r′*) and *I*_2_(*t*,*r′*) the number of infected and re-infected animals at time *t* in the other rooms *r’* belonging to the same facility, *β*_*air*_ the transmission rate per airborne route and *N*_*r′*_ the total number of growing pigs in the other rooms *r’* belonging to the same facility.

Therefore, the global force of infection *λ*(*t*,*r*), at time *t* in room *r* is:

$$\lambda \left(t,r\right)=\;\lambda_{w}\left(t,r\right)+\lambda_{b}\left(t,r\right)$$

#### Transition rates

Thirty-two transitions were considered to represent swIAV dynamics (Fig 2). Equations determining the transition rates for each health state transition (TR_n_, as shown in Fig 2) are presented in Table 3. Durations of passive and active immunity were assumed gamma-distributed and modeled using 7-exponential classes (21 transitions). Piglets with MDAs are only partially protected because of a reduction of susceptibility depending on the age of the animals. Nine health states are subjected to the transmission events depending on the host serological status (MDA-positive (7 transitions), naïve and previously-infected animals). Three transitions reflect the duration of the infectious periods depending on the infectious statuses of the animals.

**Table 3:** Equations determining the transition probabilities for each health state transition (illustrated in Fig 2) in each facility type.

Event (Transition rates TR_n_)	Equations	Animals	
		Breeding sows	Growing pigs
**MDA-positive animals**			
- Immunity waning (TR_1_ → TR_7_)	*$\frac{\sigma_{m}}{7}M^{i=\left\{1,2,\ldots ,7\right\}}{}_{}^{}$*		✓
- Infection (TR_8_ → TR_14_)	*λ*(*t*,*r*) ε *M*^*i =* {1,2,…,7}^		✓
**Naïve animals (Never infected before)**			
- Infection (TR_15_)	*λ*(*t*,*r*) *S*_1_	✓	✓
- Recovery (TR_16_)	*γ*_1_*I*_1_	✓	✓
- Immunity waning (TR_17_ → TR_23_)	*$\frac{\sigma_{1}}{7}R_{1}^{i=\left\{1,2,\ldots ,7\right\}}$*	✓	✓
**Animals susceptible to reinfection**			
- Infection (TR_24_)	*λ*(*t*,*r*) *S*_2_	✓	✓
- Recovery (TR_25_)	*γ*_2_*I*_2_	✓	✓
- Immunity waning (TR_26_ → TR_32_)	*$\frac{\sigma_{2}}{7}R_{2}^{i=\left\{1,2,\ldots ,7\right\}}$*	✓	✓

*λ*(*t*,*r*) is the global force of infection at time *t* in batch *b*, *γ*_1_ is the recovery rate for infected animals *I*_1_, *σ*_1_ denotes the immunity waning after a swIAV infection, *σ*_*m*_ is the maternal immunity waning. These rates are applied to their relative state variables representing the evolution of the individuals throughout the epidemic process (Fig 2).

#### Time steps

At time-step *t*, the time to next event *δt* is randomly drawn from an exponential distribution with expected value determined by the sum of batch-specific transition rates. The time of next event being set to *t* + *δt*, the event type is randomly chosen among all possible transitions and transition rates are updated.

#### Between-facility transmission

At each batch movement between farm facilities, susceptible animals can get infected during transfer *via* airborne indirect transmission coming from neighbouring rooms. The number of newly infected animals is randomly selected among the transferred susceptible animals (*S*_1_ and *S*_2_), following a binomial distribution with probability $1-e^{-\beta_{air}\;\pi \left(t,a\right)}$, with *π*(*t*,*a*) the prevalence of infectious individuals at time *t* in facility *a*.

#### Renewal process

At weaning, a replacement process is considered in order to replace the culled sows. The number of sows to be culled is randomly selected according to a binomial distribution with parameters the number of breeding animals in the batch and the mean culling rate per year (Table 1). Culled animals are selected independently from their infectious status. A corresponding number of susceptible gilts are then introduced in the service room, keeping a constant number of breeding sows in each batch.

#### Parametrization

All parameters involved in the infectious process are fully described in Table 2 along with their definition and the origin of the imputed values. In the service room, as the sows are reared in individual-housing system with limited contacts, the virus transmission rate was assumed to be lower than in the other facilities and was based on within-room airborne transmission experimental data [16].

### Uncertainty analysis

Parameters governing herd population dynamics are well identified [34,35] but some parameters governing the infection process are not fully known. Therefore, an uncertainty analysis was performed on unknown epidemiological parameters, also called “factors”, to investigate their effects on simulation output. Hence, a large range of values, called “levels”, were tested, as detailed in Table 2. The parameters included in this analysis are: *(i)* the between-batch transmission rate through the airborne route *β*_*air*_, *(ii)* the duration of the post-infectious immunity period (1/*σ*_1_) and *(iii)* the efficacy of immune protection after a previous challenge (*φ*) acting on the duration of the active immunity period, the duration of shedding period and the transmission rate. Using a full factorial design, three to six parameter levels have been investigated for each factor involving the 108 combinations. As the model is stochastic, 100 simulations, each of 4 years, have been run for each scenario (*i*.*e*. combination of levels).

The variation of the model behaviour according to the range of the uncertain factors has been described by several complementary and independent output variables: the number of days with shedding sows in the herd, the percentage of infected batches during the whole simulation and the average number of days with shedding piglets per batch. A multivariate linear mixed-effect model was applied to explain the variability of the selected variables with each uncertain factor included as fixed effects and simulations taken as random effect. First-order interactions were considered. Comparisons between all levels of each factor were performed using a Scheffe’ test.

### Assessment of characteristics related to swIAV within-herd persistence

The impact of maternal immunity on the duration of swIAV within-herd persistence was assessed through the analysis of two scenarios: (1) Piglets with MDAs have a similar susceptibility to swIAV infection as fully susceptible animals (*ε* = 1); and (2) Piglets with MDAs are less susceptible than fully susceptible animals (*ε* = 0.39 [16]). A survival analysis was carried out to study swIAV time to fade-out at the herd level (*i*.*e*. first time without shedding animals in breeding sows AND growing pigs). A non-parametric (Kaplan-Meier estimate) and a proportional hazard Cox model were performed to compare those two scenarios in terms of within-herd swIAV persistence. Based on models outputs for the second scenario, the duration of the epidemics within batches, age of the piglets at infection-time and number of shedding piglets at the epidemic peak were compared according to the proportion of piglets having MDAs within batch (Kruskall-Wallis global test followed by a pairwise Wilcoxon Rank Sum Test with Holm adjustment) to further explore which epidemic characteristics were strongly modified by the level of maternal immunity. All statistics were done using SAS 9.4 (SAS Institute Inc., Cary, NC, USA, 2012) and R [42].

## Results

### Effects of uncertain parameters on model outputs

For each scenario, one hundred simulations have been run, which was enough to stabilize stochastic model outputs. Increasing this number of simulations did not further reduce the variability in model outputs (data not shown).

**Fig 3:**
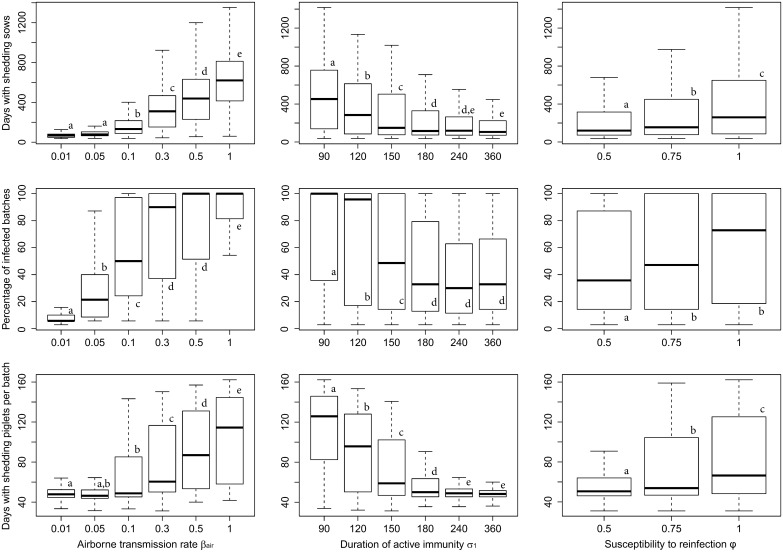
Boxplots representing the exploration of the variation of selected outputs (rows) according to the range of variation of each uncertain factor (columns). The first column corresponds to the variation of the factor “between-batch transmission rate through the airborne route *β*_*air*_”, with 6 variation levels. The second corresponds to “the duration of the active immunity period *σ*_*1*_”, with 6 variation levels and the third column corresponds to “the susceptibility to reinfection *φ*”, with 3 variation levels. Different letters within panel (a, b, c, d and e) represent statistically different distributions (Kruskal-Wallis test).

The results of the multivariate linear mixed-effect models for the three model outputs are displayed in Fig 3 (in rows) for the three fixed effects (in columns). Each boxplot represents the distribution of the output variable across all simulations with one fixed factor. The percentage of infected batches during the whole simulation increased significantly with the value of *β*_*air*_ reaching a maximum with values of 0.3 and above. The number of days with shedding piglets per batch and the number of days with shedding sows in the herd increased with incrementing values of *β*_*air*_ starting from 0.1. Increasing the duration of active immunity *σ*_1_ significantly reduced the number of days with shedding piglets per batch and shedding sows in the herd until a median 180-day plateau. If no protection was conferred after a first infection (susceptibility to reinfection *φ =* 1), the percentage of infected batches, and the number of days with shedding piglets and sows increased. Hence, an effective immune protection after a previous challenge (*φ <* 1) decreased all epidemiological output statistics because of a longer duration of active immunity (*σ*_2_), a shorter duration of shedding (*γ*_2_) and a lower transmission rate (*β*_2_).

Considering the plateaus attained for the different outputs, the parameter values were fixed to *β*_*air*_ = 0.1, *σ*_1_ = 180 days and *φ =* 0.75 for the second part of the analysis.

### swIAV endemic persistence

**Fig 4:**
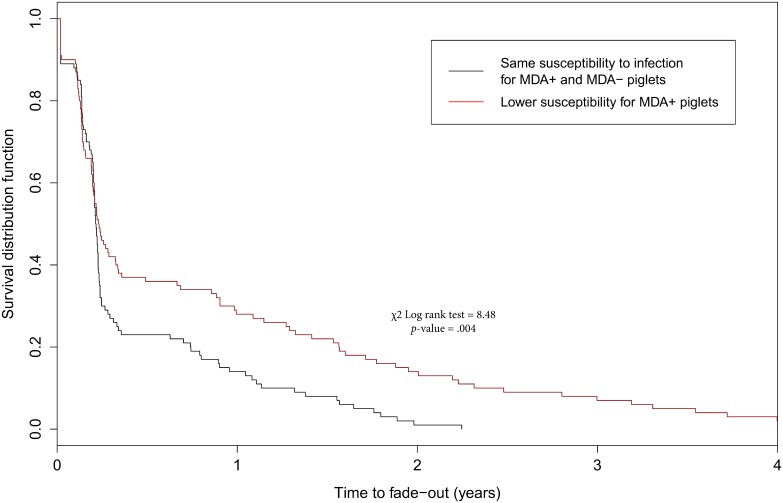
Survival analysis of swIAV within-herd persistence according to the level of susceptibility to infection for MDA-positive piglets. 100 simulations per scenario, χ^2^ Log rank test = 8.48, *p*-value = .004.

Including a lower susceptibility in MDA-positive piglets delayed the time to infection fade-out and increased the percentage of persistence at the end of the simulations compared to scenarios with an equal susceptibility for MDA-positive and negative piglets (Fig 4, χ^2^ Log rank test = 8.48, *p*-value = .004). In addition, the Cox proportional model indicated a significantly decreased risk of swIAV fade-out at the herd level if a lower susceptibility in MDA-positive piglets was considered (Hazard Ratio = 0.65 [0.48–0.87]; *p*-value = .004).

**Fig 5:**
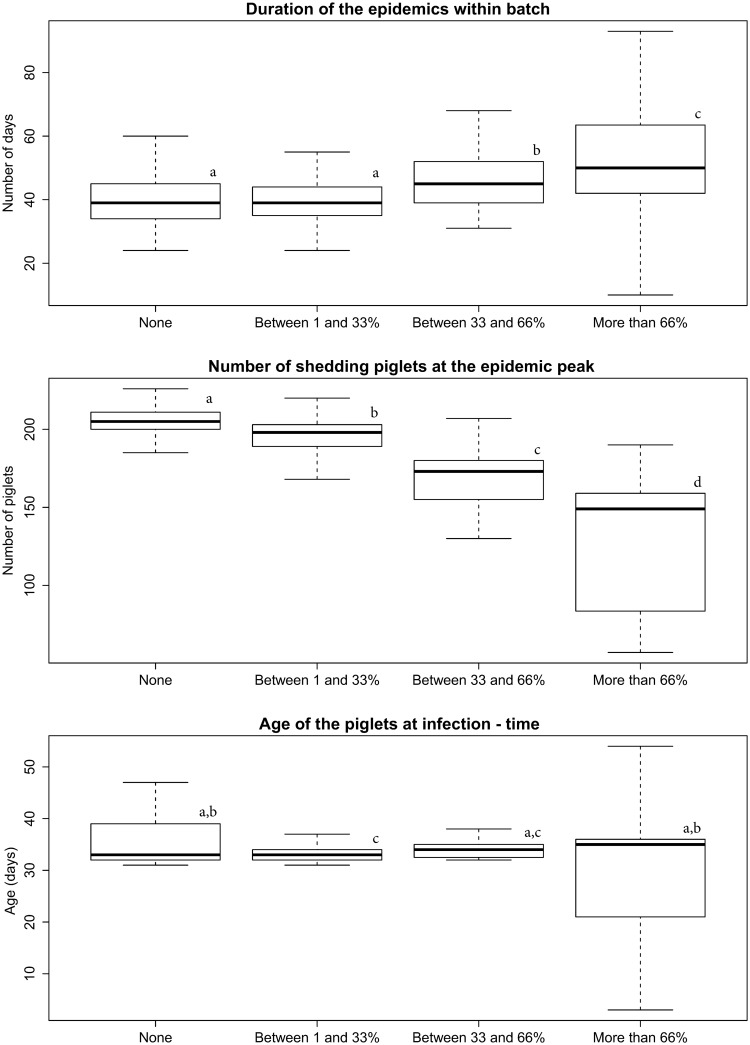
Characteristics of the epidemics within batches according to the prevalence of MDA-positive piglets. A. Duration of the epidemics within batch. B. Number of shedding piglets at the epidemic peak. C. Age of the piglets at infection-time.

To compare the characteristics of the epidemics within batches, the prevalence of MDA-positive piglets at birth was categorized as such: no MDA-positive piglets within the batch, from 1 to 33% of MDA-positive piglets within the batch, from 33 to 66% and more than 66%. The variability of the outputs observed for the “More than 66%” category was increased compared to batches with a lower proportion of MDA-positive piglets. The increasing prevalence of MDA-positive animals induced a longer duration of the epidemics and a lower number of shedding piglets at the epidemic peak (Fig 5). The age at infection-time also significantly decreased with the increasing prevalence of MDA-positive animals although the median of each category were really close (Kruskal-Wallis tests, *p*-values < .001).

## Discussion

Several studies suggested MDAs as a puzzling component for swIAV infection dynamics in pig farms [4,13]. Our model shows that the lower susceptibility observed in MDA-positive piglets [16] modifies the characteristics of the infection process, leading to a higher risk of swIAV within-herd persistence.

To our knowledge, only two modeling studies have focused on swIAV dynamics in swine populations. Reynolds et al. [25] developed two distinct deterministic models to represent swIAV dynamics in breeding and wean-to-finish farms. They found that a unique virus introduction would lead to endemicity in breeding herds whereas disease extinction was observed in wean-to-finish farms. Although they represented a realistic metapopulation structure for the breeding herd, a simple homogeneous mixing has been considered in wean-to-finish farms. This latter assumption could explain the rapid virus spread leading to disease extinction in the absence of susceptible animal introductions. In the Pitzer’s et al. stochastic model [31], swIAVs were shown to persist in populations of relatively low size probably due to the inflow of susceptible animals. The authors found a significant correlation between the herd size and the seroprevalence data in Dutch finishing herds, whereas no relationship could be evidenced in farrow-to-finish herds. These results, along with a recent risk factor analysis [43], suggest the population dynamics and the contact structure between subpopulations as important factors for swIAV transmission dynamics within herds. We therefore developed a metapopulation model accounting for the herd-location of the breeding and growing pig subpopulations to accurately represent the population dynamics and the contact structure. These population dynamics were further coupled with an epidemiological model representing swIAV infection process.

Most epidemiological parameter values were derived from published data where available; otherwise, an uncertainty analysis was performed to assess the impact of unknown parameters on the model behavior and to calibrate their values to observed swIAV endemic persistence at the herd level. Depending on the farm facilities, no quantitative information was available to estimate the between-room airborne transmission rate. Moreover, reinfection caused by the same subtype has not been observed in experimental conditions [15], possibly because of the relatively short interval between swIAV inoculations. Therefore, the between-room airborne transmission rate, the duration of immunity and the efficacy of the protection after a previous challenge (memory effect) were subject to an uncertainty analysis to assess their impact on the epidemics process.

Three complementary and independent outputs representing the herd level persistence and the infection intensity both in the breeding sows and the growing pigs were selected to investigate the swIAV epidemic characteristics (percentage of infected batches during the whole simulation, number of days with shedding sows and with shedding piglets per batch). Output analyses allowed the selection of biologically realistic values for the three unknown parameters (*β*_*air*_ = 0.1, *σ*_1_ = 180 days and *φ =* 0.75). With this parameter set, endemic persistence could be observed at the herd level throughout the simulation time and infection dynamics was consistent with observational data. Endemic persistence of swIAVs in swine operations are characterized by mechanical occurrence of epidemics at similar age (*i*.*e*. commonly around two weeks after weaning), in successive batches [4]. The shedding period at the batch scale was also found to last around a month in this observational study. Low values for airborne transmission rate (*βair* < 0.1) did not reproduce the spread of the swIAV in successive batches as observed in field conditions. In addition, values higher than 0.3 produced early and prolonged epidemics occurring around the weaning age with a huge variability between simulations (data not shown). With a transmission rate via airborne route fixed at 0.1, the median duration of the epidemics was 42 days with a median age at infection of 34 days in the present model.

A 180-day duration of active immunity, corresponding to the slaughter-age [35], appears consistent with field observations as reinfection by the same subtype during a short-time interval was deemed unlikely in growing pigs [15]. With a lower duration of active immunity, the number of days with infected piglets per batch was highly heterogeneous between successive batches (due to reinfection events) whereas homogeneity was observed from batch to batch in field conditions. At the breeding sow level, duration of active immunity shorter than 180 days induced a high number of days with shedding sows which seemed inappropriate for endemic swine flu. As no effects of a previous infection with the same subtype on the following shedding characteristics (immune memory effect) would also be surprising, a value of 0.75 has been selected because it provided consistent results on the different outputs with the selected values for the other parameters.

## Conclusion

In the present study, the maternally-derived immunity has been shown to induce a greater likelihood of persisting infection at the herd level. The presence of MDA-positive piglets leads to long-lasting epidemics within the batch, favoring transmission to new incoming susceptible piglets in neighbouring batches in intensive batch-segregated swine production systems. This increases the likelihood of herd-level endemic persistence and is essential in the design of control strategies for swIAV outbreaks in farrow-to-finish pig farms. Increasing the complexity of the model would be worth considering, to evaluate the impact of co-circulation of different subtypes, reassortment events and antigenic drift on swIAV persistence.
